# A comprehensive review of methodologies and application to use the real-world data and analytics platform TriNetX

**DOI:** 10.3389/fphar.2025.1516126

**Published:** 2025-03-10

**Authors:** Ralf J. Ludwig, Matthew Anson, Henner Zirpel, Diamant Thaci, Henning Olbrich, Katja Bieber, Khalaf Kridin, Astrid Dempfle, Philip Curman, Sizheng S. Zhao, Uazman Alam

**Affiliations:** ^1^ Lübeck Institute of Experimental Dermatology, University of Lübeck, Lübeck, Germany; ^2^ Department of Dermatology, University Hospital Schleswig-Holstein Lübeck, Lübeck, Germany; ^3^ Institute and Comprehensive Centre for Inflammation Medicine, University-Hospital Schleswig-Holstein, Lübeck, Germany; ^4^ Department of Cardiovascular and Metabolic Medicine, Institute of Life Course and Medical Sciences, University of Liverpool, Liverpool, United Kingdom; ^5^ Department of Medicine, Aintree University Hospital, Liverpool University Hospitals NHS Foundation Trust, Liverpool, United Kingdom; ^6^ Azrieli Faculty of Medicine, Bar-Ilan University, Safed, Israel; ^7^ Unit of Dermatology and Skin Research Laboratory, Galilee Medical Center, Nahariya, Israel; ^8^ Institute of Medical Informatics and Statistics, Kiel University, Kiel, Germany; ^9^ Dermato-Venereology Clinic, Karolinska University Hospital, Stockholm, Sweden; ^10^ Department of Medical Epidemiology and Biostatistics, Karolinska Institutet, Stockholm, Sweden; ^11^ Dermatology and Venereology Division, Department of Medicine (Solna), Karolinska Institutet, Stockholm, Sweden; ^12^ Centre for Musculoskeletal Research at University of Manchester, Manchester, United Kingdom; ^13^ Centre for Biomechanics and Rehabilitation Technologies, Staffordshire University, Stoke-onTrent, United Kingdom

**Keywords:** real-world data, TriNetX, cohort study, Kaplan–Meier estimator, drug discovery

## Abstract

Randomized controlled trials (RCTs) are the gold standard for evaluating the efficacy and safety of both pharmacological and non-pharmacological interventions. However, while they are designed to control confounders and ensure internal validity, their usually stringent inclusion and exclusion criteria often limit the generalizability of findings to broader patient populations. Moreover, RCTs are resource-intensive, frequently underpowered to detect rare adverse events, and sometimes narrowly focused due to their highly controlled environments. In contrast, real-world data (RWD), typically derived from electronic health records (EHRs) and claims databases, offers a valuable counterpart for answering research questions that may be impractical to address through RCTs. Recognizing this, the US Food and Drug Administration (FDA) has increasingly relied on real-world evidence (RWE) from RWD to support regulatory decisions and post-market surveillance. Platforms like TriNetX, that leverage large-scale RWD, facilitate collaborations between academia, industry, and healthcare organizations, and constitute an in-depth tool for retrieval and analysis of RWD. TriNetX’s federated network architecture allows real-time, privacy-compliant data access, significantly enhancing the ability to conduct retrospective studies and refine clinical trial designs. With access to currently over 150 million EHRs, TriNetX has proven particularly effective in filling gaps left by RCTs, especially in the context of rare diseases, rare endpoints, and diverse patient populations. As the role of RWD in healthcare continues to expand, TriNetX stands out as a critical tool that complements traditional clinical trials, bridging the gap between controlled research environments and real-world practice. This review provides a comprehensive analysis of the methodologies and applications of the TriNetX platform, highlighting its potential contribution to advance patient care and outcomes.

## Introduction

Randomized controlled trials (RCTs) are the reference standard trial design for demonstrating causality of a specific intervention, pharmacological or non-pharmacological, which includes evaluation of efficacy and adverse events (Hariton and Locascio, 2018). Usually, stringent inclusion and exclusion criteria, as well as randomization, are applied, in part for safety, but also to offset the effects of both known and unknown confounders, whilst follow-up visits and timing of visits are highly controlled (Bhide et al., 2018). These stringent criteria minimize selection bias which may impact on the translatability (Hernán et al., 2004). However, RCTs are not feasible for all research questions and as such have a number of limitations, namely,: 1. Lack of feasibility of high resource-intensive RCT for long term outcomes, 2. RCTs are often underpowered to detect harm, 3. Results of some RCTs may lack broad applicability due to their narrow eligibility criteria, relative small sample size, highly controlled implementation of interventions and comparators, and shorter duration of study. Often the focus or primary endpoint is on short-term surrogates or composite outcomes, which again may lack translatability in a real-world setting, especially for long-term outcomes. Additionally, in RCTs it is extremely challenging to address ethically challenging issues, such as the use of drugs in pregnancy or other vulnerable populations. Based on these considerations some proponents of real-world evidence (RWE) often highlight that RCTs may not be feasible, ethical, or timely (Hernán and Robins, 2016).

Unlike traditional clinical trial data, real-world data (RWD) are often generated from alternative sources often collected such as claims databases, patient generated data and most often electronic health records (EHRs). Consensus on the precise definition of RWD is lacking, however the most consistent definition is ‘data collected in a non-randomized controlled trial setting’ (Makady et al., 2017). The Food and Drug Administration (FDA), defines RWD as ‘the data relating to patient health status and/or the delivery of healthcare routinely collected from a variety of sources’ (U.S. Food and Drug Administration, 2024b). RWD is becoming increasingly important in healthcare decisions, where RCT data are lacking such as in rare diseases and off-label drug therapy, including use of drugs in vulnerable patient populations, such as during pregnancy. More recently in 2018, the FDA created a framework for the evaluation the potential use of RWE to support the approval of a new indication(s) for a drug already approved under the Federal Food, Drug, and Cosmetic Act and to help support post-approval drug study requirements. Importantly, RWD harbors significant potential for generation of RWE but also notably for designing and conducting subsequent confirmatory studies including RCTs. RWE through RWD analysis may answer questions that may not be otherwise addressed (Liu and Panagiotakos, 2022). Furthermore, RWD can serve as an alternative to clinical cohort studies for assessing potential risk factors that cannot be randomized, such as patient characteristics (e.g., comorbidities, blood type, ethnicity) or lifestyle factors that are either ethically (e.g., smoking) or practically (e.g., long-term diet) unfeasible to randomize. This approach is particularly relevant for studying population risks, which conceptually cannot be examined in RCTs.

TriNetX (TriNetX LLC, Cambridge, MA, USA) is an extensive growing global network expanding from 55 healthcare organizations (HCOs) and 7 countries in 2017 (Palchuk et al., 2023) to 137 HCOs and 17 countries as of August 2024. It is often described as a real-world ecosystem given its real time continually updated nature. The platform is a federated network which facilitates collaboration between industry including pharmaceutical companies and research organizations, academia, HCOs and community-based HCOs. TriNetX has a security and governance model that provides a federated platform of RWD (electronic health records, datasets) which is Health Insurance Portability and Accountability Act (HIPAA), General Data Protection Regulation (GDPR), and Lei Geral de Proteção de Dados (LGPD)-compliant.

The aim of this paper is to review the methodologies, applications and published studies using the real-world data and analytics platform TriNetX.

## Setting the stage: A brief introduction to the TriNetX platform

Given the challenges of sustainability of academia governed and financed clinical data repositories, encompassing granular electronic health records (EHRs), the private company TriNetX has successfully implemented an alternative approach for the funding and operational management of clinical data repositories in 2014. Here, industry partnerships with healthcare organizations (HCOs) led to the establishment of a large federated clinical data repository. In this setting, TriNetX operates the platform (including the legal framework and analytical tools), funding is provided by industry, and HCOs provide EHRs (Palchuk et al., 2023). From this cooperation, industry and academia can access over 150 million EHRs as of September 2024. Industry participants usually use the federated TriNetX database to query patient counts to challenge feasibility of in- and exclusion criteria for clinical trials. These patient counts can be queried globally, restricted to certain regions, or to single HCOs. This enables real-time iteration and the optimization of trial design and patient recruitment that has recently been described and reviewed in-depth elsewhere (Palchuk et al., 2023; Topaloglu and Palchuk, 2018; Claerhout et al., 2019; Campion et al., 2024). Conversely, the focus of this review is on the methodologies and application of the TriNetX network for epidemiological research. Access to the TriNetX is either by commercial licensing agreement (industry) or by collaboration agreement (academia).

**FIGURE 1:**
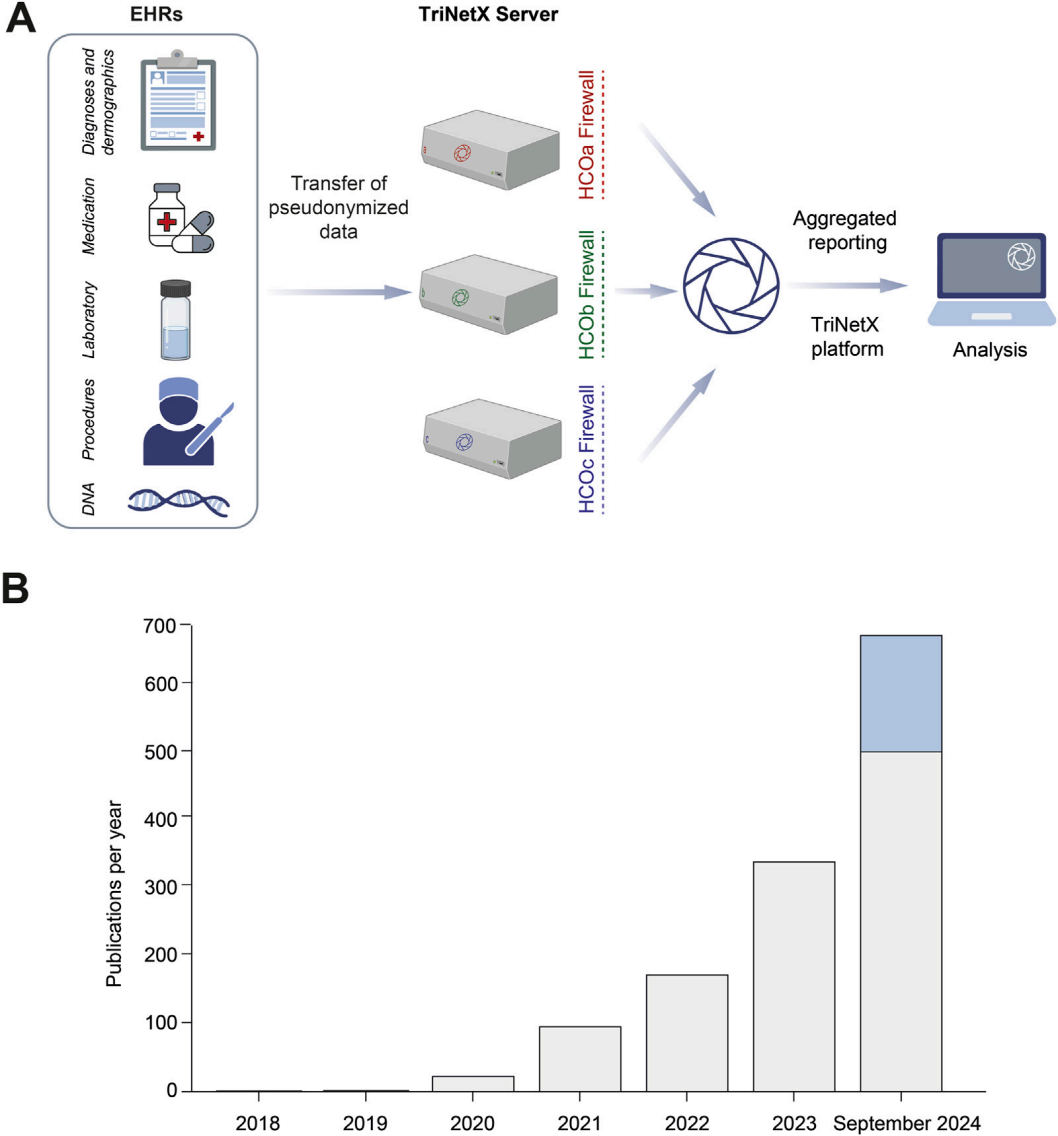
Structure of and number of publications with the TriNetX platform. **(A)** Electronic health records (EHRs) encompassing longitudinal data on demographics, diagnoses, medication, laboratory results, procedures and (limited) genetics (depicted as DNA) are provided by each of the TriNetX cooperating healthcare organizations (HCOs). Data from the HCOs’ EHR system are pseudonymized and then transferred to a TriNetX server that is located behind the HCO firewall. Analysis by an end-user accessing the TriNetX platform only allows to analyze aggregated data from several HCOs. **(B)** Number of publications using or referring to the TriNetX platform. In 2018, two manuscripts using TriNetX were published. This number increased to three in 2019, and more rapidly thereafter, reaching close to 300 from January to June 2024 (depicted in grey). Based on this, we assume close to 700 publications using the TriNetX platform in 2024, with another steep increased in the subsequent years.

As depicted in Figure 1A, at each HCO pseudonymized EHR data are transferred from the own clinical data management system to a server that is still behind the HCOs’ firewall. If a query is made outside the HCO, only aggregated data from several HCOs can be retrieved. Thus, the retrieved datasets only contain de-identified data as per the de-identification standard defined in the Health Insurance Portability and Accountability Act (US federal law) Privacy Rule (TriNetX. Publication Guidelines, 2017). As such, studies with TriNetX typically do not require evaluation by an Ethical Review Board. Within the TriNetX network, EHRs can be retrieved and analyzed from five Collaborative Networks: The Global Collaborative Network (as of September 2024, 153.5 million EHRs from 128 HCOs), the US Collaborative Network (117.2 million EHRs from 66 HCOs), the Latin America (LATAM) Collaborative Network (31.5 million EHRs from 32 HCOs), the Europe, Middle East, and Africa (EMEA) Collaborative Network (19.2 million EHRs from 26 HCOs), and the Asian Pacific (APAC) Collaborative Network (5.1 million EHRs from 14 HCOs). In addition, data from the HCO that one is affiliated with can be retrieved. The US and Global Collaborative Networks can be used with or without natural language processing (NLP). NLP allows to use data from unstructured documents to extract standardized information, such as medications and diagnoses. The Collaborative Networks are, with the exception of the Global Collaborative Network, mostly distinct and do not contain overlapping data. Since the establishment of this infrastructure in 2014, research on the platform has rapidly increased: In 2018, two articles were published that were authored by TriNetX employees. The article by Dr. Stapff compared cardiovascular outcomes in patients treated with either sodium-glucose co-transporter-2 (SGLT2) inhibitors or dipeptidyl peptidase 4 inhibitors and demonstrated lower risks in those treated with SGLT2 inhibitors (Stapff, 2018). The article by Drs. Topaloglu and Palchuk reviewed the use of the TriNetX network to optimize clinical trial operations (Topaloglu and Palchuk, 2018). Since then, the number of publications using the TriNetX network has dramatically increased. In total, as of September 2024, there close to 1,000 publications, with 457 of these published between January and September 2024 (Figure 1B). These include publications in high-ranking journals such as Nature Medicine (Wang et al., 2024a), Lancet Rheumatology (Singla et al., 2023), and Lancet Psychiatry (Taquet et al., 2022). Overall, this indicates the high relevance of real-world data for biomedical research.

### In more detail: Study design, data retrieval and analysis on the TriNetX platform

**FIGURE 2:**
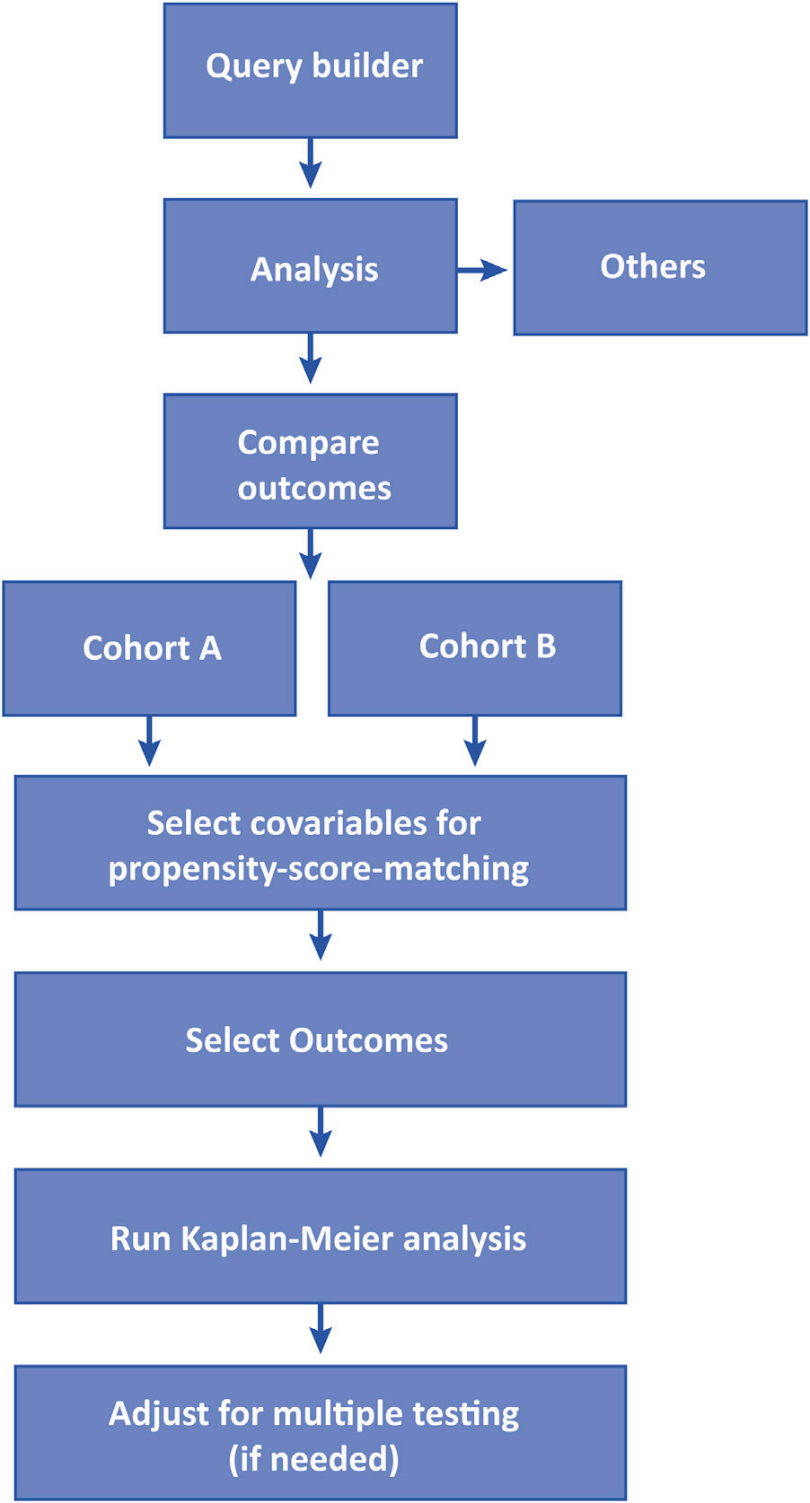
Workflow of the analytical steps using TrinetX.

The TriNetX platform enables users to conduct retrospective studies by accessing deidentified EHRs. The retrospective study design and analysis tool are user-friendly, guiding users step-by-step to build a cohort and analyze outcomes. Initially, users create individual cohorts using a query builder, and in the subsequent step, these cohorts are analyzed for outcomes of interest (Figure 2).

#### Query Builder

To initiate an analysis, users select the relevant Collaborative Network (Global, US, Latin America, “Europe, Middle East, and Africa”, or Asia Pacific) from which EHR data will be retrieved. Cohort construction then involves applying specific inclusion (“must have”) and exclusion (“cannot have”) criteria, with at least one inclusion criterion originating from the “diagnosis” category. Criteria are grouped according to international coding systems, including “demographics,” “diagnosis” (ICD-10-CM, ICD-9), “oncology” (ICD-O, AJCC, NAACCR, TNX curated), “procedures” (ICD-10-PCS, CPT, HCPCS, SNOMED), “medications” (VA, ATC), “labs” (LOINC), “genomics” (Gene), and “visits”. When multiple criteria are applied, they can be linked using the Boolean operators “and” (requiring all selected criteria to be present in the same individual) or “or” (requiring the individual to meet at least one of the criteria). Each criterion can be further refined with filters, such as specifying an age range for a diagnosis or a route of administration for a medication. Additional details can be integrated into each group, including the number of occurrences, unique instances, creating related groups, or adding temporal constraints to define relationships between groups. Multiple groups can be used to define patient cohorts, and these groups can also be connected using the Boolean operators “and” or “or”. This approach is particularly useful for selecting patients with a specific diagnosis within a certain timeframe after an initial procedure, or for including individuals with predefined baseline characteristics. Time constraints can also serve as an alternative to the built-in tool to exclude outcomes before the index event (referring to a specific occurrence and/or point in time used to define the starting point for follow-up in the cohort). If more than one group of definitions is used to define a cohort, the index event must be chosen from among them. Once the cohort is defined, users can explore all cohort characteristics using the “explore cohort” function, which includes demographics (e.g., age, sex, ethnicity), diagnoses, oncology, procedures, medications, labs, and genomics. These characteristics encompass all data reported by healthcare organizations (HCOs) at any given time. The platform also provides additional tools following the query builder, including “analyze criteria,” “rate of arrival,” “summary statistics,” and “analysis,” although the latter three tools may be of limited use for most academic users.

#### Analytics

Once the study cohort(s) have been constructed, by default, the analytics tool currently offers six distinct analysis options: “Analyze Outcomes”, “Compare Outcomes”, “Compare Cohorts”, “Treatment Pathways”, “Incidence and Prevalence”, and “Advanced Explore Cohort”. There are also additional analytical tools under development that may be accessible for beta-testing. Among these, “Compare Outcomes” is the most commonly used function for research purposes. While other options such as “Treatment Pathways” and “Advanced Explore Cohort” may also provide valuable insights, their applicability can be limited by data availability and ease of use. Similar considerations apply to the “Treatment Pathway Analysis” that has only reported in few publications (Ghosh et al., 2024; Henao-Martínez et al., 2023). Therefore, the focus is often on the “Compare Outcomes” function, though the general methodology can be extended to the other functions.

**FIGURE 3:**
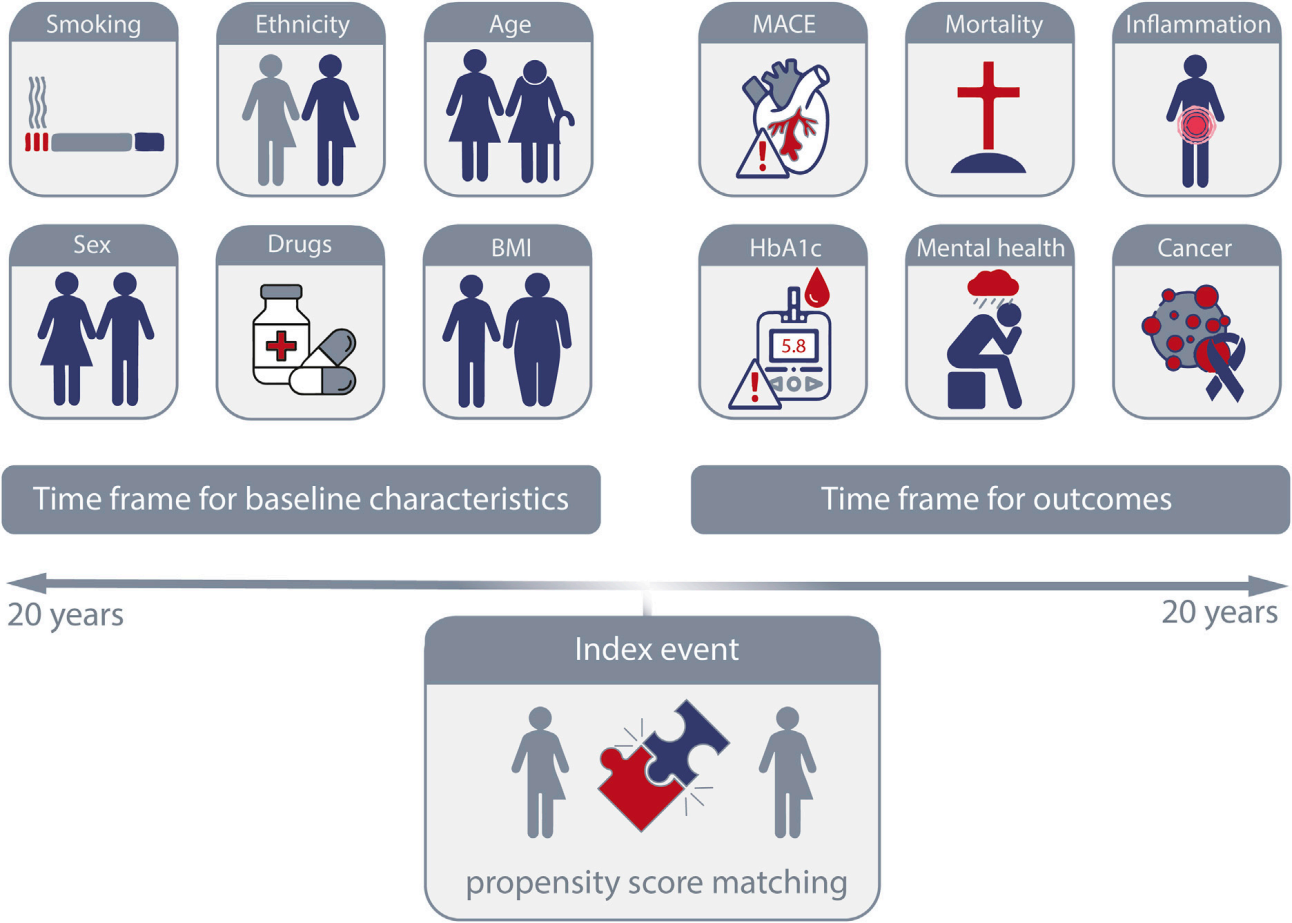
Definition of index event, baseline characteristics and outcomes. On the TriNetX platform, the index event that is defined by a specific occurrence or point in time to define the starting point for follow-up is defined by the selected criteria for each cohort. Baseline characteristics of the cohorts occurring at any time (up to 20 years) prior to the index event can be used for propensity-score matching or for an in-depth description of the cohorts. Outcomes following the index event, again for up to 20 years after the index event, are by default considered 1 day after the index event.

In the “Compare Outcomes” function, users select two predefined cohorts from the query builder to compare. The platform then guides users through a structured five-step analysis process, organized around three time segments: Index event, time prior to the index event, and the period following the index event (Figure 3). For each cohort, an index event is defined, such as the first diagnosis of a disease or the initial treatment with a specific drug after diagnosis. This index event corresponds to the baseline in prospective studies. The three time segments include the period before the index event (up to 20 years), the index event itself (on the same day), and the period following the index event (up to 20 years). After defining the index event and timeframe for the first segment, both cohorts are evaluated for differences in characteristics (present before the index event) that could serve as potential confounders for the outcomes of interest. These key characteristics are then used as criteria for propensity score matching at the index event. The criteria for matching typically include “Demographics”, “Diagnoses”, “Oncology”, “Procedures”, “Medications”, “Labs”, and “Genomics.” Baseline characteristics are analyzed before matching using the “Baseline Comparison Statistics” function, which checks for differences between the unmatched cohorts and ensures that the matching criteria are appropriate. In the “Balance Cohorts” step, users finalize the propensity score matching by selecting input variables and reviewing the matching results. In the final segment, two analysis functions are available: “Explore Outcomes” and “Outcomes”. The “Explore Outcomes” function serves as a preliminary screening tool, identifying statistical differences between cohorts without performing matching. Outcomes marked during this step are further analyzed in the “Outcomes” function, where users refine the analysis by setting the timeframe after the index event (e.g., 1 day to 3 months or 1–5 years) and confirming the index event. The outcome analysis can be conducted using one of four available methods: “Measures of Association” (risk ratio, risk difference, odds ratio with 95% confidence intervals, t-test), “Kaplan-Meier Analysis” (log-rank test, hazard ratio), “Number of Instances” (t-test), and “Lab Results” (t-test for numeric values, chi-square test for categorical values). As a recent addition, follow-up time for each cohort can also be retrieved.

For the first three analysis methods, it is recommended to exclude patients who experienced the outcome event before the index event. This exclusion can be enabled in the “Options” section. The final results are provided in the “Results” section, and the complete analysis, including network details, inclusion and exclusion criteria, matching characteristics before and after matching, analysis methods, and individual outcome analyses, can be downloaded.

### Statistical considerations

#### Sample size and power analysis

In prospective studies, sample size calculation should be performed before data collection to ensure adequate power (“as many as necessary”) while keeping cost and potential risk to participants low (“as few as possible”). For retrospective studies, sample size may be limited by the availability of data and an *a priori* analysis of statistical power should ensure that a planned investigation is adequately powered to detect meaningful differences or associations (Kim and Seo, 2013). If such minimal power requirements are fulfilled, all available data should be used in retrospective studies and there is no need to decrease sample size to only obtain, e.g., 80% power. To perform a power calculation, the specific test (e.g., log rank test, chi-square test, t-test) and the anticipated effect sizes (e.g., hazard ratio, proportions, standardized mean difference) as well as the desired significance level need to be specified. For this, free online power calculators and dedicated programs are available, such as G*Power (Heinrich Heine Universität Düsseldorf, 2020) or in R, the “powerSurvEpi” package may be used to calculate the power for survival analysis based on the Cox proportional hazards model (CRAN.R-project, 2021). Generally, the very large sample size available in the TriNetX platform offers high power even for rare exposures (index events) and for rare outcomes. A key advantage of utilizing all available data in retrospective studies is the ability to generate more precise effect estimates. However, concerns regarding “overpowering” may arise. Overpowering, however, mostly relates to prospective study designs where excessive sample sizes may lead to detecting statistically significant but clinically trivial differences. In retrospective analyses, the interpretation of results should focus on clinical significance, ensuring that findings are not only statistically meaningful but also relevant to patient outcomes and clinical decision-making.

#### Propensity score matching (PSM)

To alleviate confounding and allow for a better baseline comparability of groups, PSM should be used. For PSM, the TriNetX platform utilizes the user-defined set of covariates for each patient within the cohorts. Using logistic regression, the probability that a patient belongs to a particular cohort is calculated. This predicted probability, known as the propensity score, reflects the probability that a subject will receive an exposure based on their covariate profile. Once propensity scores are generated, the system tries to match each patient from the smaller cohort with a corresponding patient from the larger cohort who has a sufficiently similar propensity score. This matching process results in a subset of both cohorts, where the distribution of covariates is balanced between the groups, and aims for less confounded comparison and analysis of outcomes between the two cohorts. This method is widely used in epidemiological and clinical research to reduce bias in observational studies, where randomization is not feasible. PSM may also be advantageous over conventional multiple Cox proportional hazards analysis (Elze et al., 2017) if there is a large number of potential confounders. If performed on the platform, the number of covariates for PSM is limited based on sample size due to computational restrictions. Hence, for PSM considering many covariates, sample sizes need to be reduced. This can be achieved by adding time windows, limiting the age range, or adding additional terms, e.g., exclusion of deceased patients.

#### Correction for multiple testing

Correcting the significance level for multiple testing in epidemiological research aims to control the family wise error rate, reducing the likelihood of type I errors - incorrectly rejecting a true null hypothesis (i.e., finding a false positive). In large-scale epidemiological analyses the chance of detecting false positives increases as the number of tests increases. Multiple testing corrections, such as the Bonferroni correction or less conservative alternatives such as the false discovery rate (FDR) correction, potentially ensure that any significant findings are more likely to reflect true associations rather than random chance. In contrast, Dr. Rothman advocates that no adjustments for multiple comparisons are necessary in epidemiological research (Rothman, 1990). While adjusting for multiple comparisons reduces type I errors, it simultaneously increases the risk of type II errors, leading to potentially missed true associations. In Dr. Rothman’s view, the underlying “universal null hypothesis,” which attributes findings to chance, contradicts the empirical research principle that nature operates under discoverable laws. By avoiding such adjustments, researchers may achieve more accurate interpretations of real-world data and feel more empowered to explore potentially significant findings without the constraints of overly conservative corrections (Rothman, 1990). Ultimately, the decision of how stringently, i.e., selecting Bonferroni or FDR, to adjust for multiple comparisons in epidemiological research should be guided by the specific context and objectives of the study. While corrections can prevent false positives, they may also obscure true associations, especially in exploratory research. Therefore, researchers should weigh the trade-offs and consider the balance between rigor and discovery in their analytical approach.

#### Odds (OR) or hazard ratio (HR)

Analyzing RWD, both odds ratios (OR) and hazard ratios (HR) can be used to compare the likelihood of events between two groups. However, they differ fundamentally in their consideration of time. OR provide a static measure, comparing the likelihood of an event occurring between two groups without taking temporal information into account. This renders OR suitable for cross-sectional and case-control studies where timing is not a factor. In contrast, hazard ratios incorporate the element of time, making them ideal for survival analysis or time-to-event studies where censoring is present, i.e., not every person experiences the event during the available follow-up time. HRs compare the rate at which events occur over time between groups and can establish whether the event occurs significantly earlier in one group compared to the other.

#### Considering the proportionality assumption

If HR are used, the assumption of proportional hazards must be met, meaning that the hazard rates between groups follow the same trend over time, differing by a constant factor (the HR). If this assumption is violated, the HR can be misleading. The TriNetX platform incorporates a test for proportionality, that was developed by Grambsch and Therneau (Grambsch and Therneau, 1994), which relies on the scaled Schoenfeld residuals. The Schoenfeld residual for a parameter reflects the difference between the observed value for a patient and the expected value based on the model, adjusted for variability through scaling by the variance. Interpreting the resulting chi-square and p-value require careful consideration, as a non-significant p-value does not prove that the assumption is fulfilled while at the same time even small deviations from this assumption (which may not relevantly affect the model) may lead to nominally significant p-values due to the large sample size. TriNetX advises users to treat these metrics as qualitative guides rather than exact quantitative measures (TriNetX, 2024). Specifically, users are encouraged not to view a significance level of 0.05 as a strict cutoff for decision-making. Instead, chi-square values should be interpreted broadly, with larger values indicating relevant deviations from proportionality while small values suggest that the proportionality assumption may hold. If there is evidence that the proportionality assumption is seriously violated, an analysis based on ORs can still be performed.

#### Importance of sensitivity analyses

To mitigate potential bias and confounding based on the selection of covariates for PSM and the outcomes, it is recommended to perform several sensitivity analyses by conducting the same analysis across different cohort variations. This helps to assess the robustness of the findings and reduces the risk that results are unduly influenced by specific cohort characteristics or assumptions. Sensitivity analyses can provide greater confidence in the validity of the study’s conclusions by testing the consistency of outcomes under different conditions. Examples for cohort variations are.

• Excluding time periods shortly after index event helps mitigate the risk of including outcomes that may not be directly related to the exposure or intervention. Early outcomes might be due to existing conditions rather than the event or intervention under study. The length of such time periods to exclude should be based on subject matter knowledge on the minimum time for the event of interest to develop. By excluding these time periods, the analysis focuses on outcomes more likely attributable to the exposure.
• Including a minimal baseline time window of patient records before inclusion in the study better ensures that there is adequate data to assess the patient’s condition before the index event. This allows for better control of confounding factors and ensures that the baseline characteristics are well-defined, improving the accuracy of the propensity score matching and subsequent analyses.
• Use of alternative covariates for PSM may result in varying levels of balance between groups, and thus allows to determine whether findings are robust across different scenarios or if they are sensitive to the specific variables selected. This ensures that the matching process captures a wide range of relevant factors, minimizing bias and enhancing the credibility of the results. It also provides a more comprehensive assessment of the potential confounders, making the findings more reliable and generalizable to different contexts.
• Excluding outcomes occurring before the index event at data retrieval instead of after PSM (which is the default option in TriNetX) better mitigates bias from influencing the matching process. If outcomes are excluded after PSM, the matching may be biased by the presence of those outcomes. Excluding them earlier in the process ensures that the matching is based purely on baseline covariates, leading to more valid comparisons between cohorts. Taking this into account, excluding outcomes before the index event prior to PSM may be better suited as a primary analysis, and the default option in TriNetX as a sensitivity analysis.
• Modifying the strictness of cohort definitions allows to explore the impact of varying levels of participant selection on study outcomes. A more lenient definition might include a broader population, enhancing generalizability but potentially introducing more variability or distortion. Conversely, a stricter definition can focus on a more specific population, improving internal validity but possibly limiting the applicability of the findings to broader groups. Selecting this for a sensitivity analysis helps determine whether the study’s conclusions are robust across different definitions or if they are highly dependent on specific criteria.
• Similar considerations apply when changing outcome definitions (more lenient or stricter).
• Changing the time window of data retrieval enables evaluation of the impact of different time frames on study outcomes. A wider time window might capture more events, potentially increasing statistical power but also introducing variability from long-term changes in treatment practices or patient characteristics. Conversely, a narrower time window focuses on more recent or immediate outcomes, which may reduce noise but limit the number of events available for analysis.

### Identification of risks in populations

**FIGURE 4:**
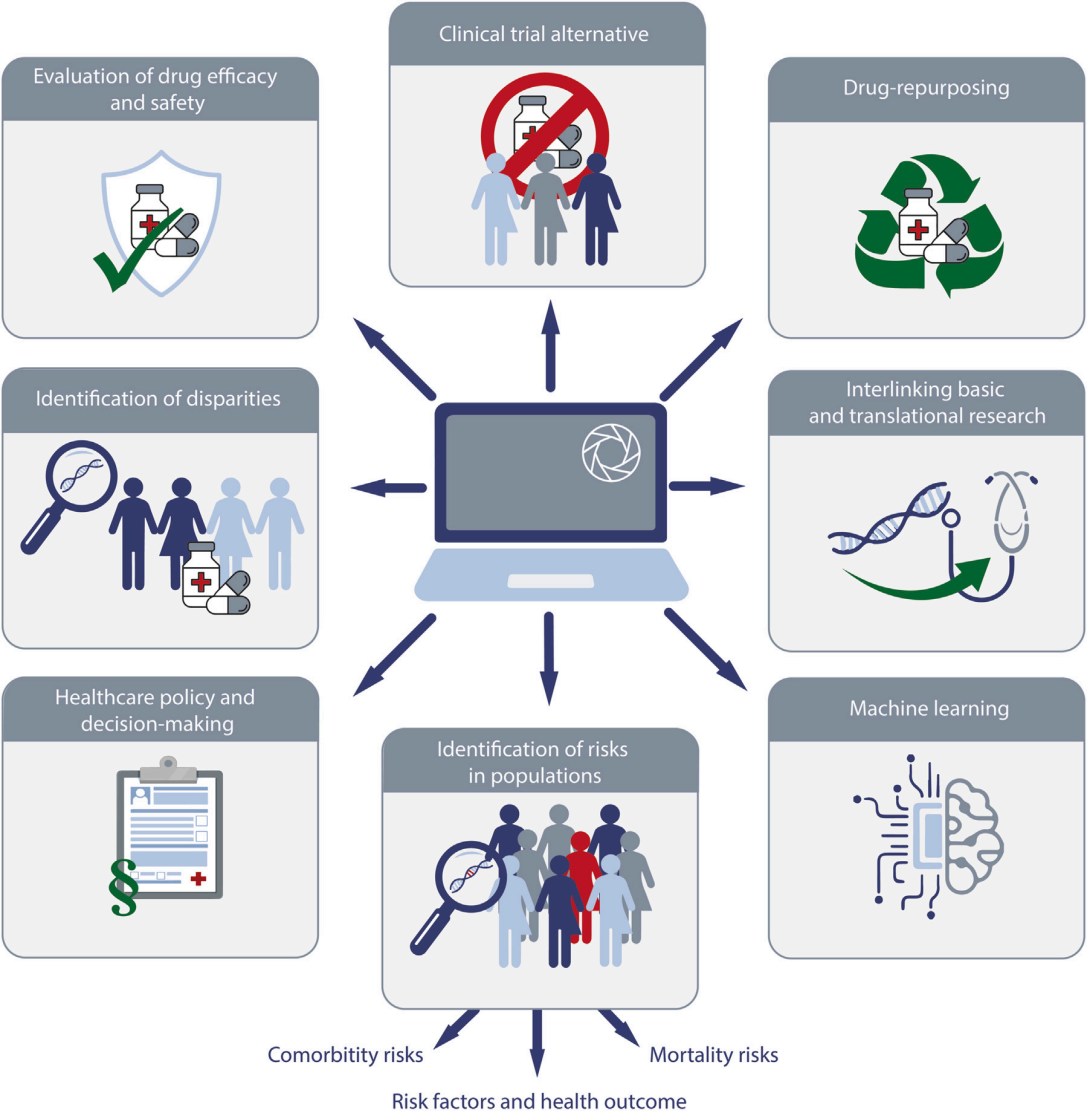
Schematic overview of the research enabled by real-world data (RWD) exemplified by TriNetX. The diagram highlights key areas of application, including (i) alternatives for randomized controlled clinical trials, (ii) drug-repurposing, (iii) interlinking basic and translational research, (iv) machine learning, (v) identification of risks in populations, (vi) providing data for healthcare policy and decision making, (vii) identification of racial disparities, and (viii) evaluation of drug safety and efficacy.

The use of real-world data platforms like TriNetX has significantly enhanced our ability to identify and analyze risks within various populations (Figure 4). This involves assessing risk factors, comorbidities, and mortality across diverse demographic and clinical groups. The ability to access a vast dataset allows for more comprehensive studies, leading to better understanding and management of health risks.

#### Risk factors and health outcomes

Understanding the myriad risk factors that contribute to health outcomes is crucial for effective disease prevention and management. The TriNetX platform, with its extensive RWD, enables researchers to identify and analyze these risk factors across diverse populations, uncovering critical insights that inform clinical practice and public health strategies. One example is the identification of pruritus (chronic itching) as a risk factor for severe health conditions. Studies have shown that pruritus can be associated with an increased risk of hematological malignancy and autoimmune diseases (Deng et al., 2022; Raap et al., 2023; Caldas et al., 2023). These findings underscore the importance of recognizing seemingly minor symptoms as potential indicators of more severe underlying health issues.

#### Comorbidity risk

Assessing comorbidities within patient populations is crucial for understanding the interplay between different health conditions and their combined impact on patient outcomes. The TriNetX database facilitates extensive analysis of comorbid conditions, offering valuable insights into how these conditions coexist and influence each other. For example, a retrospective cohort study using TriNetX data revealed an increased risk of autoimmune diseases in patients who had recovered from COVID-19, highlighting the long-term health implications of the virus (Chang et al., 2023). Similarly, another study examined long-term cardiovascular outcomes in COVID-19 survivors, finding significant risks associated with the virus in non-vaccinated populations (Wang et al., 2022). Other examples of studies focused on comorbid health conditions utilizing the TriNetX platform include the investigation into the co-existence of various autoimmune disorders among patients with pre-existing autoimmune blistering diseases (Kasperkiewicz et al., 2023) and comorbid risk of cardiovascular outcomes in patients with systemic lupus erythematosus (Olbrich et al., 2023) or prurigo nodularis (Olbrich et al., 2024). These studies emphasize the importance of monitoring patients for comorbid conditions to ensure comprehensive healthcare management.

#### Mortality risk

Understanding mortality risk is a key component of population health management. The TriNetX platform’s extensive dataset enables researchers to conduct in-depth analyses of factors contributing to mortality, aiding in the development of strategies to mitigate these risks. For example, research utilizing TriNetX demonstrated that statin use is associated with a reduced risk of liver disease, hepatocellular carcinoma, and liver-related mortality (Vell et al., 2023). Similarly, another study comparing treatments for acute stroke, such as tenecteplase *versus* alteplase, have identified significant differences in mortality and bleeding complications, informing clinical decision-making (Murphy et al., 2023). All-cause mortality in patients with type 2 diabetes undergoing different treatments (Riley et al., 2023) and the mortality risk in Stevens-Johnsons syndrome and toxic epidermal necrolysis managed in different ways (Ozhathil et al., 2024) have been investigated using the TriNetX platform. Matched case-control studies for mortality among different conditions can also be performed (Boch et al., 2023). These types of investigations into different aspects of mortality risk provide critical insights into the nature of different diseases and the effectiveness of various treatments and their impact on patient survival.

#### Where clinical trial data are lacking

We have previously detailed that RWD can provide RWE where RCT data are lacking, e.g., investigation of rare (adverse) events, head-to-head comparisons, or off-label medications, or, in particular, where RCTs are unlikely to occur do to cost implications. Recently, Anson et al. published a RWD study utilizing the TriNetX platform of multimodal pharmacotherapy of a combination of metformin, pioglitazone, sodium-glucose-linked-cotransporter-2 inhibitor (SGLT2i), and glucagon-like peptide-1 receptor agonist (GLP-1RA) *versus* a more conventional glucocentric treatment (insulin and sulphonylureas) for cardiovascular outcomes in the treatment of type 2 diabetes (Anson et al., 2024a). The pathophysiology of type 2 diabetes is complex including β cell failure, muscle, and liver insulin resistance, increased renal glucose reabsorption, reduced incretin response, α cell insulin resistance, reduced secretion of and increased resistance to GLP-1 and glucose-dependent insulinotropic polypeptide (GIP) and appetite dysregulation (DeFronzo, 2009). As such, this paper assessed the real-world evidence in which combination therapy targeted these pathophysiological mechanisms. It demonstrated that intensive combination therapy, targeting the distinct pathophysiological defects in type 2 diabetes was associated with favorable cardiorenal outcomes, compared to conventional insulin and sulphonylurea therapy. Additionally, a potent GLP-1 RA, namely, semaglutide was reassessed for a link to suicidal ideation, however a large TriNetX Network analysis (240,618 patients) did not demonstrate higher risks of suicidal ideation with semaglutide compared with non-GLP1R agonist anti-obesity or anti-diabetes medications (Wang et al., 2024a). Importantly previous RCTs have been too small and of too short duration to effectively answer this important question necessitating RWD to generate RWE was required to provide reassurance to patients and clinicians to the widely used pharmacotherapy. Similarly in the dermatology space, Kridin et al. demonstrated the putative negative effect of oral corticosteroid vs. topical corticosteroid for bullous pemphigoid on mortality at 1 and 3 years (Kridin et al., 2024a). Such cardiovascular and mortality outcome studies are unlikely to ever be undertaken in an RCT setting but are essential to capture the long-term impact of treatment outcomes.

More recently, the specific methodology of target trial emulation has been used to apply the principles of RCTs to observational data (Matthews et al., 2022). This approach can help estimate the causal effect of an intervention and has several key steps which include; 1. Designing a hypothetical RCT, 2. Specify the protocol including eligibility criteria, treatment strategies, outcomes, etc., and 3. Utilizing the available observational data to emulate the hypothetical RCT. Such methodology can be utilized with the TriNetX platform. Recently, Wang et al. (Wang et al., 2024b), demonstrated that semaglutide was associated with lower risks for tobacco used disorder-related healthcare measures in patients with comorbid T2DM and tobacco use disorder compared with other anti-diabetes medications including other GLP-1RAs. This study utilized target trial umlauted approach which specifically improves the quality of observational studies investigating the effects of interventions by providing a structured framework to help overcome the limitations of observational studies and improve causal inference.

### Evaluation of drug effectiveness and safety

TriNetX data can also serve as a resource for evaluating both the effectiveness and safety profiles of pharmaceuticals currently employed in clinical practice. Given the absence of disease severity documentation within the network, surrogate markers of effectiveness must be relied upon. While possible exceptions exist, such as the assessment of vaccination efficacy using the corresponding infectious disease as an outcome, this approach has not, to our knowledge, been utilized with EHR data from TriNetX. Additionally, known sequelae of a given disease, such as psoriatic arthritis in relation to psoriasis, or mortality risks, can serve as indicators of drug efficacy (Singla et al., 2023). To evaluate drug efficacy or safety, the study should retrieve EHRs with an ICD10-code for the disease of interest, e.g., acne vulgaris. Patients with this condition are then allocated to distinct groups based on their exposure to one of two possible treatments. Whenever feasible, treatments should be chosen based on their position within the treatment hierarchy, allowing for comparison of outcomes between two same-line treatments. Otherwise, the risk of “confounding-by-indication” (in this context failure of first line treatment) might be high and this cannot be controlled by the propensity score matching described above as the relevant characteristic (previous first line treatment) will mostly be present in one group. TriNetX has been widely used to study drug efficacy and safety. Examples are the safety of isotretinoin use in acne vulgaris and biological treatment of chronic inflammatory diseases, that are described in more detail below.

#### Safety of isotretinoin in acne vulgaris treatment

Acne is a chronic inflammatory disease of the pilosebaceous unit, driven by immune responses affecting adolescents and young adults. Clinically, it manifests as both non-inflammatory lesions, i.e., comedones, as well as inflammatory lesions, such as papules, pustules, nodules, and cysts. Predilection sites are the face, chest, and back (Moradi Tuchayi et al., 2015). Oral antibiotics, especially tetracyclines are commonly used first line treatments for severe acne (Costa et al., 2021). Another first line treatment of severe acne is oral isotretinoin (Reynolds et al., 2024). However, the use of isotretinoin in patients with acne has been associated with potentially serious risks, including the potential development of psychiatric disorders and inflammatory bowel disease (Chen et al., 2022; Taylor et al., 2023). Yet, the risks of psychiatric disorders and inflammatory bowel disease in patients with acne exposed to oral isotretinoin have remained a matter of debate (Wright et al., 2021; Tan et al., 2024). With TriNetX, large cohorts of in patients with acne exposed to isotretinoin or antibiotics can be retrieved. This allows the investigation of a substantial number of EHRs along with extensive propensity-score matching for potential confounding factors for the risk to develop psychiatric disorders or inflammatory bowel disease. Regarding psychiatric disorders, a recent study using data from TriNetX, demonstrated that isotretinoin is associated with a lower risk of depression, post-traumatic stress disorder, anxiety, bipolar disorder, schizophrenia, and adjustment disorder compared to oral antibiotics. The risk of major depressive disorder and suicidal attempts were similar between the groups, although the risk of suicidal ideation was higher with isotretinoin (Kridin and Ludwig, 2023a). This finding has recently been replicated in a meta-analysis of 24 studies (Tan et al., 2024), underscoring the validity of the results obtained from TriNetX. Risk of Crohn’s disease or ulcerative colitis (UC), in patients with acne exposed to either oral isotretinoin or oral antibiotics was explored in a recent study which reported no increased lifetime risk of CD or UC in the isotretinoin group compared to the antibiotics group. In a time-restricted analysis of the same dataset, a transient increase in UC risk during the first 6 months of isotretinoin use was observed, with five additional cases per 10,000 patients, but this risk diminished when later time points were investigated. Interestingly, the risk of irritable bowel syndrome, that was used as an additional endpoint, was lower in patients exposed to isotretinoin (Kridin and Ludwig, 2024; Kridin and Ludwig, 2023b).

#### Biological treatment of chronic inflammatory diseases

Biological treatment has become an invaluable treatment for many non-communicable, chronic inflammatory diseases and several malignancies (U.S. Food and Drug Administration, 2024a). Data from clinical trials relating to these compounds, however, span a limited time frame. Hence, additional benefits or previously unforeseen adverse events, e.g., reactivation of tuberculosis following TNF inhibition (Solovic et al., 2010), are likely to be overlooked. In this context, RWD can substantially enhance the assessment of drug safety and effectiveness. One landmark paper in this context has been published on the risk of inflammatory arthritis in patients with psoriasis treated with different classes of biologics (Singla et al., 2023). Psoriasis is a chronic inflammatory disease primary affecting the skin (Griffiths et al., 2021) that is associated with a high extracutaneous comorbidity, such as depression, psoriatic arthritis, metabolic disorders and cardiovascular disease, of which psoriatic arthritis is most frequent (Gershater et al., 2024). The aforementioned study compared the risk of developing psoriatic or inflammatory arthritis in psoriasis patients who were treated with tumor necrosis factor inhibitors (TNFi), interleukin-17 inhibitors (IL-17i), or IL-12/23i. Here, patients treated with IL-12/23i had a significantly lower risk of developing psoriatic or inflammatory arthritis compared to those treated with TNF inhibitors. Given these findings are validated in prospective studies or data from prospective registries, this would have a significant impact on the management of psoriasis (Singla et al., 2023). Other studies in psoriasis using TriNetX contrasted the risk of infections (Kridin et al., 2023a), malignancies (Kridin et al., 2024b), and cardiovascular diseases (Kridin et al., 2023b) in psoriasis patients treated with different groups of biologics. In line with the findings regarding the risk of psoriatic or inflammatory arthritis, the risk of infections was lower in patients treated with either IL17-or IL12/23-inhibitors, as opposed to those managed by TNFi (Kridin et al., 2023a). Similar findings were made regarding the risk of malignancies (Kridin et al., 2024b). Contrasting the risk of cardiovascular diseases in psoriasis patients treated with either IL17-or IL12/23 inhibits found no significant differences (Kridin et al., 2023b).

#### Predicting treatment outcomes

The inhibition of immune checkpoints, which are negative regulators of immune activation that normally limit antitumor responses, has led to almost unparalleled clinical responses in patients with various types of cancer (Ribas and Wolchok, 2018). However, immune checkpoint inhibition is potentially complicated by (cutaneous) immune-related adverse events. These are often mild to moderate, are treatable and reversible. In some cases, immune-related adverse events can lead to lasting damage and death (Ramos-Casals et al., 2020). Cutaneous immune-related adverse events or presence of certain autoantibodies causing these, may indicate a positive response to immune checkpoint inhibition, and improved patient survival (Hasan Ali et al., 2020). To address this assumption in more detail, a retrospective cohort study used data from TriNetX, retrieving patients with cancer treated with immune checkpoint inhibition who developed cutaneous immune-related adverse events and matched with patients who did not. Development of cutaneous immune-related adverse events within 6 months after initiation of therapy was analyzed, adjusted for demographics, cancer type, and stage. Presence of cutaneous immune-related adverse, such as pruritus, drug eruption, xerosis, nonspecific rashes, psoriasis, and lichen planus/lichenoid dermatitis, were associated with a reduced mortality risk. The authors concluded that cutaneous immune-related adverse are strongly linked to better treatment responses and improved survival (Tang et al., 2022).

Taken together, these studies demonstrate that TriNetX is a useful tool for retrospectively analyzing drug effectiveness and safety. It is important to note that the lack of established causality must be considered when interpreting this data, in particular potential confounding by factors not available for consideration in the propensity score matching. In the interim, however, these insights serve as a valuable resource for identifying potential risks and guiding treatment selection.

### Drug-repurposing

Investigation of drug effects in diseases outside their approved indications can accelerate and facilitate drug development for otherwise neglected diseases. Drug-repurposing strategies include *in silico* predictions on drug pathways or *in vitro* and animal investigations, but also observational studies. RWD can aid the identification of potential repurposing candidates, and several use cases have demonstrated beneficial effects of drugs previously unrelated to a certain disease. In the field of cancer research, TriNetX helped evaluate survival rates in patients with head and neck cancer and beneficial effects of metformin (Gaertner et al., 2024) and statins (Wüster et al., 2023) were found. By contrast, other drugs such as ibuprofen showed unfavorable results in this regard (Ebeling et al., 2024). These negative findings are, however, equally important as those that demonstrate beneficial effects. Specifically, the findings of a reduced survival of ibuprofen-exposed patients with head and neck cancer, compared to non-exposed patients urges caution when selecting pain medication in these patients. In patients with breast cancer, also, statins reduced the risk of all-cause death (Bucci et al., 2024). Further, several drug analyses supported by TriNetX were conducted for neuropsychiatric diseases given the orphan status and insufficient interventional options in many of them. For neurodegenerative diseases, reduced prevalence of Alzheimer’s disease (Silva et al., 2023) and Parkinson’s disease (Silva et al., 2024) was found in patients treated systemically with calcineurin inhibitors. Likewise, in a study which received significant public attention, a reduced risk of dementia was observed in persons exposed to the recombinant shingles vaccine (Taquet et al., 2024). Reduced frequencies of sleep and anxiety disorders were documented in patients treated with brain-penetrant calcium channel blockers (Colbourne and Harrison, 2022; Colbourne et al., 2021). Also, outcomes in several substance abuse disorders were significantly affected by drug-repurposing candidates: A drug screen identified eight medications that led to more frequent remissions in patients with opioid use, including psychiatric drugs as well as propranolol and prazosin (Patel et al., 2023). Cannabis use disorder and relapses were less frequent in patients treated with semaglutide (Wang et al., 2024c). Most notably, ketamine was identified as an efficient treatment for cocaine use disorder by first employing an AI-based screening strategy, expert review, and subsequent RWD confirmation in the TriNetX database (Gao et al., 2023a). The same prediction algorithm was also used to identify drugs that delay the progression of cataract in patients with diabetes mellitus (Gao et al., 2023b). Recently, a study comparing off-label tirzepatide use in people living with obesity but without T2D to those treated with semaglutide use reported a significant reduction in incident risk of developing T2D and serves as a vital hypothesis generating foundation for future definitive RCTs (Anson et al., 2024b). Taken together, TriNetX is a powerful tool to gather retrospective evidence for the effects of repurposing candidate drugs, however, confirmatory clinical trials are necessary.

### Identification of ethnical disparities

RCTs may lack ethnic variation with the overrepresentation of White participants in particular (Kaakour et al., 2022). Although there have been efforts to recruit underrepresented minority ethnic groups, the lack of RCT data has resulted in a clear evidence gap in which drug effect may differ due to underlying ethnicity-specific biological mechanisms. In the NICE guidance for type 2 diabetes [NG28], a specific research recommendation was based on determining the ’effectiveness of SGLT2 inhibitors for different ethnic groups’. Taking this forwards, Anson et al. completed a real-world study of 98,094 participants propensity score matched (1:1). The subsequent analysis demonstrated that Black (regarding mortality and ischemic heart disease) and Asian (regarding heart failure and hospitalization) individuals demonstrate a greater degree of benefit compared to White individuals (Anson et al., 2023). When considering dermatological conditions, Kasperkiewicz et al., using data from the TriNetX platform, demonstrated that while the risk of mortality is comparable between White and skin of color (SOC) patients with immunobullous diseases, SOC patients have a significantly higher risk of hospitalization and post-inflammatory hyperpigmentation (Kasperkiewicz et al., 2025). Hypotheses generated were either higher rates of disease activity or inequality in access to healthcare. Clearly, these data need translating into studies of ethnicity-orientated disease mechanisms or healthcare access. Other RWD studies of dermatological conditions, namely, prurigo nodularis, a chronic inflammatory skin disorder which is intensely pruritic and has nodules of a hyperkeratotic nature on the torso and extremities, has also shown ethnic disparities in Black patients. There was greater mortality and possible systemic inflammation in Black patients (Sutaria et al., 2022). Although, these RWD demonstrate association rather than causality, they provide adequate data for the development of future RCTs.

### Provide data for decision-makers to set healthcare policies

RWD provides decision makers with a second pillar of evidence, including information on treatments, therapy pathways, and outcomes which can be stratified by baseline demographics and clinical characteristics. RWD can offer reassurance to clinicians and the wider public that evidence borne out in RCTs is (or sometimes is not), applicable to real world populations. RCTs have strict inclusion and discontinuation criteria, but the practice of clinical medicine is highly heterogenous, and many therapies can, and are prescribed to patients who do not necessarily fully satisfy the inclusion criteria of the study where the drug was initially investigated in. RWD includes data from a broader range of patients, capturing variations in age, gender and comorbidities; allowing decision makers to understand how interventions work across diverse groups, leading to more inclusive and effective policies. By analysing outcomes stratified by certain baseline characteristics, decision-makers can promote a personalised medicine approach to their populations, ultimately improving clinical outcomes of patients. RCTs are limited by the lack of longer term follow up, and some beneficial and harmful outcomes of interventions are only captured in the extension phase of RCTs, which are often not done. RWD can follow patients over extended periods, often over decades, providing insights into the long-term safety and efficacy of treatments. RWD is often live, and continuously updated. The agility of such data ecosystems allows decision makers to react quickly to emerging health issues and novel therapies, subsequently allowing the timely adaptation of policy to respond to new threats. Finally, where the disease of interest is rare, or the intervention novel, or not widely used in a subset of the population, RWD can highlight areas where existing evidence is lacking, provide supplementary rationale to support novel RCTs, and guide future research investment, particularly in populations and therapies that may otherwise be overlooked. This use case of the TriNetX platform was especially useful during the COVID-19 pandemic, where RWD addressed critical questions within a short time-frame (Kovvuru et al., 2021; D’Silva et al., 2021; Taquet et al., 2021; Rivera-Caravaca et al., 2021).

### Linking machine learning and TriNetX

Within TriNetX, the LUCID feature enables researchers to utilize complex statistical tools and machine learning *in situ*, enabling the ability to download data while decreasing the time it takes to explore whether the data is fit for the research question proposed. RWD has been used to develop and validate a risk prediction model for pancreatic ductal adenocarcinoma. Using a large dataset of over 1.5 million cases and controls, 87 different features ranging from pre-existing diagnosis, medication and demographic data were harnessed. Authors were able to internally and externally validate their model; and simulate deployment of their model for early prediction of 6–18 months prior to diagnosis. The model captured over 3x more patients at risk of pancreatic ductal adenocarcinoma compared to existing screening programs of individuals at similar risk levels (Jia et al., 2023). Additionally, RWD has informed research evaluating different equations and methods of estimating glomerular filtration rate (GFR) in children, with further research required to confirm these findings which otherwise, without large populations to study, would go unasked, and ultimately, unanswered (Dagenais et al., 2024).

### Interlink with basic and translational research

Integrating RWD into larger research questions enables the foundation of basic and translational research to be firmly rooted in practical, everyday experiences. More specifically, RWD allows to generate hypotheses and validate mechanisms observed in basic research. Conversely, RWD may also be used to confirm findings from basic or translational research, thus providing a holistic understanding of biological processes across diverse settings. Ultimately, RWD serves as a vital bridge between fundamental research and real-world applications, enriching the overall research landscape. This interlink of basic and translational research with RWD is an emerging field, with, so far, few publications in the field. As an example, using RWD to confirm and expand upon translational research findings, researchers investigated the molecular effects of anti-tumor necrosis factor (TNF) treatment on vaccination responses. This was prompted by recent findings questioning whether vaccination of patients with chronic, non-communicable inflammatory diseases under TNF-targeting treatment leads to impaired vaccine-induced immune responses and protection against breakthrough infections. The study used COVID-19 vaccination to investigate IgG subclass levels, IgG-Fc glycosylation patterns, B cell subsets, and antibody effector functions in patients on anti-TNF or other immunosuppressive treatments. Using TriNetX, the risk of SARS-CoV-2 breakthrough infections in these patients was assessed. Anti-TNF treatment reduced long-term levels of all anti-spike protein IgG subclasses with low galactosylation and sialylation. Initially, reactivated memory B cells produced highly galactosylated and sialylated IgG antibodies, which declined after each booster, especially in the elderly. Reduced IgG1 levels in these anti-TNF treated patients were linked to lower functional activity and a higher risk of COVID-19 (Buhre et al., 2025).

### Strengths and limitations of RWD

RWD has become increasingly valuable in healthcare, offering significant insights beyond traditional clinical trials. The volume of data captured by some ecosystems can exceed over 150 million individuals, enhancing the reliability of any findings described using such datasets. Data generated from RWD is more applicable to the lived experience of individuals and can be seen as ‘RCT-affirming’ studies, applicable to real world clinical practice. RWD often include the individual over many years, sometimes for most of their life. Such repositories of longitudinal data allow analysis of significantly large populations to track longer term health outcomes and treatment adherences, otherwise seldom investigated by other means, or if so, come at great expense and inconvenience. Research using even the largest of databases is highly cost-effective and more resource efficient than conducing new, large, multi-centre clinical trials. Additionally, novel treatment regimens can be analysed using RWD and be put forward as hypothesis generating, exploring associations that may have otherwise not gained the interest of sponsors or industry.

The limitations on utilizing RWD must be acknowledged. Firstly, by the very nature of the data source and lack of randomization, confounding variables, if unaccounted or unadjusted for, can bias any results generated using RWD. Importantly, causality cannot be established with observational data, only correlation. If RWD spans multiple countries, or multiple health institutions, variability in practice, coding for diagnosis and management of conditions can lead to high degrees of heterogeneity in data, and if stratification by location is not possible, may complicate interpretation and generalizability of findings. Besides variability in practice at the nation or healthcare system level, intra-caregiver variability can subject RWD to further inconsistencies, coding errors and missing data, further compounding the accuracy and reliability of such data sources. Incorporating and homogenizing unstructured data inputted from various settings can be challenging, and lead to further inaccuracies. Although RWD incorporates a larger more diverse population pool compared to RCTs, for RWD that originate from economies with large health, and access to health inequalities, such as the United States, underserved and overlooked communities may still be underreported. To overcome many of the confounding variables as described, complex and sometimes technically advanced statistical analysis is required to balance cohorts, which may lead to data attrition. Additional limitations that apply to most RWD databases, including TriNetX, are lack of confidence in drug exposure, lack of documented disease severity and missing information on patient reported outcomes.

### Strengths and limitations of the TriNetX platform

As of September 2024, access to over 150 million EHRs is provided by TriNetX for academic research. This large, and continuously growing number of EHRs allows to investigate rare diseases such as epidermolysis bullosa acquisita (Kridin et al., 2023c) or hereditary angioedema (Sylvestre et al., 2021). Another advantage of the large dataset is the possibility to account for bias stemming from confounding factors, by use of extensive propensity score matching. Furthermore, this also allows to perform several sensitivity analyses, by changing the investigated time periods, adding minimal baseline time windows, generation of age-, sex-, or ethnicity-stratified groups, as well as changing definitions of index event and outcome definitions. From the TriNetX networks that can be accessed by default, this applies to the Global and the US Collaborative Network, as each of these encompass well over 150 million EHRs. The other Networks contain, in most cases, too few EHRs to allow investigation of rare diseases or an in-depth propensity score matching. In line with this notion, in the US Collaborative Network, the provided data is highly granular, i.e., demographics, diagnoses, medications and procedures are well coded. In other networks, e.g., the LATAM Collaborative Network some diagnoses seem underreported, exemplified by 0.02% EHRs indicating nicotine dependence within a total of over 20 million EHRs. Based on surveys, this proportion is expected to be around 11% (Andrade et al., 2002). Another example that relates to coding practices is the racial/ethnicity documentation in the EMEA Collaborative network. Here all EHRs are documented with an Unknown Ethnicity, and close to 85% are documented with an Unknown Race.

While the Global and US Collaborative Networks provide data for detailed research, other networks have limitations in certain areas. The high granularity of data required for specific analyses is primarily restricted to the US, as the Global Network is predominantly composed of US data. These gaps are not attributed to the TriNetX database but rather result from to the legislation concerning data protection and coding practices in countries outside the US. Adopting data protection policies and documentation requirements globally would significantly improve the data quality within TriNetX. Such improvements would enable more comprehensive and detailed studies across diverse populations and regions. Addressing these documentation and policy issues could unlock the full potential of RWD within and outside the TriNetX platform, establishing an unparalleled resource for global health research.

Within TriNetX a comprehensive and easy to use analytical pipeline is embedded. Amongst the seven analyses provided by default, the Compare Outcomes function is most commonly used. This and the other provided analyses are described in detail above (Setting the stage: Study design, data retrieval and analysis on the TriNetX platform). Overall, the navigation and running the analysis is very intuitive. At completion of the analysis all results can be downloaded. Depending on local legislation and access rules investigators may be able to download additional data. The default analyses available on the TriNetX platform effectively address the majority of research questions commonly explored. Indeed, most of the publications using the database, used this analytical tool. However, legislation concerning data protection also limit research with the TriNetX platform outside the US. More specifically, download of large datasets is not possible. This is, for example, needed to apply more complex analytical tools, e.g., AI, to determine predictors of the disease (Jia et al., 2023). Within the US it is also possible to build, and train own analytical models. This, for example, allows to cluster patients for safety and efficacy analyses, as well as more refined statistical analyses, e.g., use of the non-parametric test (Mann-Whitney) instead of t-test for laboratory values, which are often skewed. This feature would also enable to re-identify patients from the HCO of the researcher for closer monitoring and/or in-depth molecular analysis. A limitation that is related to the analytical pipeline itself is the missing option to build more complex outcomes. Currently, one or more outcomes can only be joined with an *or* denominator. It is currently not possible to implement time constraints or other more complex outcome definitions. For some diseases, especially those that cannot directly be coded with an ICD10-code alone, a more complex definition of outcomes would significantly add to the analytical pipeline embedded on the TriNetX platform.

If treatments are investigated on the platform, these must be considered as intention-to-treat analyses in most cases because drug compliance is not systematically captured on the platform. In more detail, to ensure that a given drug is prescribed for the investigated indication, a respective time-constrain has to be implemented when retrieving the data. This increases the likelihood that this drug was prescribed for the indication under investigation. However, compliance is not documented and can only be assumed using proxies. For example, by analyzing the number of events of drug prescription following the index event. Alternatively, a second (or third) documentation of drug prescription after a certain time period following the index event can increase the likelihood of continued drug exposure. If pseudonymized datasets are accessible (on a large-scale, currently only in the USA) this can be analyzed in more detail. However, drug compliance can only be documented when the same HCO documents the prescription.

In the past, TriNetX has continuously increased the number of EHRs available on the platform by expanding the network of contributing HCOs within existing networks. In addition, otherwise underrepresented areas are increasingly in the focus of TriNetX, exemplified by the rapidly growing LATAM Collaborative Network that mainly include EHRs from Brazil. Furthermore, the analytical platform has been extended. These additions to the analytical pipeline were, to a major part, driven by the request of the researchers using the platform. Specifically, follow-up time is provided for most analyses since 2024, and the Cox Proportional Hazard Models has recently been added to the analytical pipeline. Several other analyses are under development, such as patient clustering, burden of illness and logistic regression.

Taken together, the TriNetX platform is an ideal instrument to address clinically highly relevant research questions. The main disadvantage relates to legislation and coding practices, rather than platform-inherent limitations. Therefore, changing legislation regarding data protection outside the US would significantly promote real-world-data research. As the TriNetX platform already is already among the leading platforms, research using TriNetX would benefit exponentially from this change in legislation.

## Conclusion

In conclusion, RCTs remain the cornerstone for establishing causality in clinical research. However, their limitations, including narrow eligibility criteria, resource intensity, and focus on short-term outcomes, restrict their applicability in broader, real-world settings. RWD not only complement RCTs but also extend beyond them by offering critical insights from more diverse populations and enabling the exploration of longer-term outcomes. Furthermore, RWD can address questions that RCTs are not equipped to answer, particularly in areas where traditional trials are impractical or unethical. This includes studying rare diseases, assessing off-label drug use, and evaluating interventions in populations often underrepresented in clinical trials. By capturing the complexities of real-world clinical practice, RWD provides a broader and more comprehensive perspective on treatment effectiveness and safety, ultimately filling the gaps left by RCTs. As the importance of RWD grows, further advancements in data governance, analytical tools, and global standardization will be crucial for maximizing its potential. Ultimately, the integration of RWD and RCTs holds the promise of more comprehensive and inclusive evidence generation, driving improvements in patient care and healthcare policy.
